# “Smart” Polylactic Acid Films with Ceftriaxone Loaded Microchamber Arrays for Personalized Antibiotic Therapy

**DOI:** 10.3390/pharmaceutics14010042

**Published:** 2021-12-26

**Authors:** Ekaterina A. Mordovina, Valentina O. Plastun, Arkady S. Abdurashitov, Pavel I. Proshin, Svetlana V. Raikova, Daniil N. Bratashov, Olga A. Inozemtseva, Irina Yu. Goryacheva, Gleb B. Sukhorukov, Olga A. Sindeeva

**Affiliations:** 1 Science Medical Center, Saratov State University, 83 Astrakhanskaya Str., 410012 Saratov, Russia; voplastun@gmail.com (V.O.P.); dn2010@gmail.com (D.N.B.); inozemtsevaoa@mail.ru (O.A.I.); goryachevaiy@mail.ru (I.Y.G.); 2 Center for Neurobiology and Brain Restoration, Skolkovo Institute of Science and Technology, 3 Nobel Str., 143005 Moscow, Russia; a.abdurashitov@skoltech.ru (A.S.A.); pavel.proshin@skoltech.ru (P.I.P.); g.sukhorukov@skoltech.ru (G.B.S.); 3 Saratov Hygiene Medical Research Center of the FBSI «FSC Medical and Preventive Health Risk Management Technologies», 1A Zarechnaya Str., 410022 Saratov, Russia; matiz853@yandex.ru; 4 Department of Microbiology, Virology, and Immunology, Saratov State Medical University, 112 Bolshaya Kazachia Str., 410012 Saratov, Russia; 5 School of Engineering and Materials Science, Queen Mary University of London, Mile End Road, London E1 4NS, UK

**Keywords:** prolonged release, ultrasound-induced release, drug delivery, microchamber arrays, PLA, antibiotics, ceftriaxone, bacteriological activity, bacterial infections, *Staphylococcus aureus*

## Abstract

Bacterial infections are a severe medical problem, especially in traumatology, orthopedics, and surgery. The local use of antibiotics-elution materials has made it possible to increase the effectiveness of acute infections treatment. However, the infection prevention problem remains unresolved. Here, we demonstrate the fabrication of polylactic acid (PLA) “smart” films with microchamber arrays. These microchambers contain ceftriaxone as a payload in concentrations ranging from 12 ± 1 μg/cm^2^ to 38 ± 8 μg/cm^2^, depending on the patterned film thickness formed by the different PLA concentrations in chloroform. In addition, the release profile of the antibiotic can be prolonged up to 72 h in saline. At the same time, on the surface of agar plates, the antibiotic release time increases up to 96 h, which has been confirmed by the growth suppression of the *Staphylococcus aureus* bacteria. The efficient loading and optimal release rate are obtained for patterned films formed by the 1.5 wt % PLA in chloroform. The films produced from 1.5 and 2 wt % PLA solutions (thickness—0.42 ± 0.12 and 0.68 ± 0.16 µm, respectively) show an accelerated ceftriaxone release upon the trigger of the therapeutic ultrasound, which impacted as an expansion of the bacterial growth inhibition zone around the samples. Combining prolonged drug elution with the on-demand release ability of large cargo amount opens up new approaches for personalized and custom-tunable antibacterial therapy.

## 1. Introduction

Bacterial infections remain a serious problem in medicine due to the high risk of numerous infectious complications accompanying injuries and surgical interventions even in developed countries [1]. In hot and humid climates, even slight skin damage without timely antibiotic therapy can lead to blood poisoning, gangrene, and amputation [2]. The development of chronic wound bacterial infections [3,4] and implant-associated infections [5,6] is often accompanied by the formation of biofilms, which leads to virulence. Currently, most biofilm remediation strategies involve developing biofilm dispersing agents to disrupt the biofilm cell community or biofilm inhibiting agents from preventing the early stages of biofilm formation [7].

Widely used antibiotics-elution materials (PMMA-based spacers [8] and beads [9]) in clinical practice have made it possible to increase somewhat the effectiveness of the treatment of acute infections in recent years [10,11]. Despite tremendous research efforts, the problem of preventing such infections remained unmet medical needs. The intensive development of biomaterials provides many alternative approaches to prevent bacterial infections [12,13,14,15,16]: nanoparticles [14,15,17,18], micro- and nanocapsules [19,20], and hydrogels [21]. However, these materials have not yet found their application in the clinic for prolonged prophylaxis and therapy, largely due to the ineffective encapsulation of low molecular weight antibiotics and insufficient drug release time (only a few hours [22,23,24,25]). In addition, the dynamic of bacterial infections and overall diseases course is individual for each patient; thus, therapy must be adjusted accordingly. It may include prophylactic goals with a low drug dosage or intensive local therapy with controlled release of a large amount of drug. In this regard, the biomaterial should perform on-demand release of the encapsulated substance to rapidly increase the local antibiotic concentration in the tissues when the clinical picture changes. The most promising material is polymer film with microchamber arrays (MCAs).

MCAs were initially introduced using a layer-by-layer method to fabricate thin film on surface modulated templates. Various polyelectrolytes were explored for MCA build-up, such as poly (allylamine) hydrochloride [26,27,28], poly (sodium 4-styrenesulfonate) [26,27,28], poly (diallyl dimethylammonium chloride) [28], poly (acrylic acid) [28], and their various combinations. Polyelectrolyte-made MCAs have drawbacks due to difficulties loading and retaining water-soluble molecules. The use of hydrophobic polymers deposited by a single layer dip-coating approach for MCA fabrication was proposed [29]. The exploitation of water-insoluble polymers, such as PLA [29,30,31], poly(lactic-co-glycolic acid) (PLGA) [31,32], polycaprolactone (PCL) [31,33], resulted in significantly faster, simpler, and scalable approach enable to entrap and retain water-soluble molecules. PLA polymer-made MCAs exhibit sufficient mechanical properties to keep structural parameters avoiding unnecessary deformation, facilitate loading due to smooth surface, and improve cargo retain compared to PCL and PLGA [31]. In vitro and in vivo studies have shown that such PLA-based MCAs are completely biocompatible and biodegradable [29,34,35]. MCAs are suitable for carrying a wide range of substances with different molecular weights, in particular, enzymes [30], polysaccharides [36], fluorescent dyes [37], neurotransmitters [34], hormones [31,32], and growth factors [35]. The studies have shown that polymer film with MCAs is sensitive to external stimuli such as ultrasound [29,30,32,38], magnetic field [39], and laser irradiation [30,36,40,41] if additional modifiers are used during their fabrication (magnetic nanoparticles and photoabsorbing agents, respectively). The choice of trigger for drug release in real medical practice must exclude a negative effect on drug load and consider material localization. The ultrasound has a high penetrating ability and does not affect encapsulated drugs. It has been shown that ultrasound may stimulate the release of antibiotics from various materials, beads, and bone cement and prevent the development of bacterial infections [42,43,44,45]. Thus, the sensitivity of MCAs to ultrasound makes it possible to control the release of the drug and individually select the therapy regimes. This promising “smart” biomaterial can be used as a dressing or coating to modify the surface of implants and stents [31,46] to provide mechanical protection, prolonged and controlled drug release.

Here, we demonstrate that the films with microchambers array based on PLA biopolymer can be used for ceftriaxone encapsulation. Ceftriaxone is a well-known antibiotic with a broad-spectrum activity against Gram-positive and Gram-negative aerobic and some anaerobic bacteria [47]. In this study, we investigated how the PLA concentration affects the release profile of ceftriaxone and its bacterial effect on *Staphylococcus aureus* (*S. aureus*). *S. aureus* is a major pathogen that causes a wide range of clinical infections. Over the past two decades, *S. aureus* infections were among the leading causes of complications after implantation devices and an epidemic of skin and soft tissue infections associated with resistance to certain antibiotics [46].

## 2. Materials and Methods

### 2.1. Materials

Polylactic acid (PLA, 3 mm granule) was obtained from GoodFellow (Huntingdon, UK). Chloroform and sodium chloride were obtained from Sigma-Aldrich (Darmstadt, Germany). Mueller-Hinton Agar No. 2 was obtained from HiMedia Laboratories (Einhausen, Germany). For the solution preparation, the deionized (DI) water (electric conductivity ∼18.2 MΩ m^−1^ at 25 °C) prepared by the Milli-Q Plus185 from Millipore (Darmstadt, Germany) water purification system was used. Antibiotic—ceftriaxone sodium salt (Ceftriaxone) was obtained from pharmaceutical company ZAO LEKKO (Volginsky, Russia). The polydimethylsiloxane (PDMS) kit (Sylgard 184) was purchased from Dow-Corning (Midland, MI, USA).

### 2.2. Fabrication of Patterned PDMS Stamp

The patterned PDMS stamp was preliminarily made based on a Kapton master for the fabrication of film with microchamber arrays. Kapton master mold was manufactured using the laser ablation method. Cobolt Tor XS (532 nm, 50 µJ, 1.9 ns) was used as a light source combined with a focusing lens (Olympus 4x/0.1 n.a.). Twenty-five laser pulses were used to form one well. Resulted wells are in a cone form with a diameter of 21 ± 1 µm, a height of 20 ± 2 µm, and an angle of ~35°. The step between wells was 40 µm. PDMS stamp was produced by the casting method. Two PDMS compounds were mixed in a manufacturing prescribed ratio (10:1 *w/w*) and poured onto the surface of the master mold. Curing was performed using the oven at 90 °C for 1 h. After complete curing, PDMS was detached from the Kapton master and gold-sputtered. After this, a new batch of uncured PDMS was poured onto the gold-coated PDMS cast surface, followed by the same curing process. The resulting PDMS stamp, which replicates features of the Kapton master, was used for patterned film manufacturing.

### 2.3. Fabrication of Microchamber Arrays Containing Ceftriaxone

**Figure 1:**
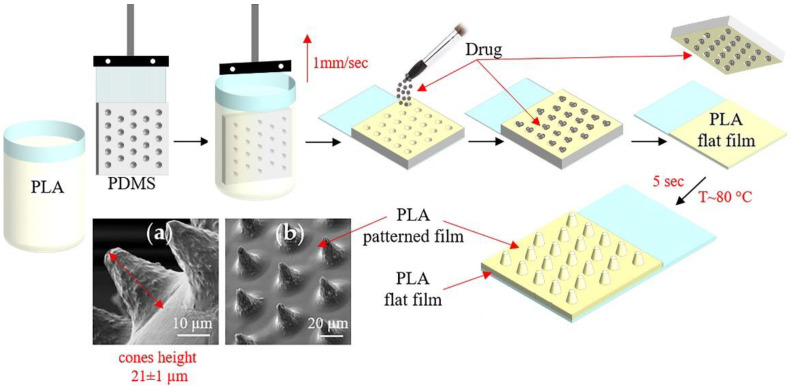
The scheme of fabrication microchamber arrays containing ceftriaxone. SEM images of the outer surface of microchamber arrays based on PLA (**a**,**b**).

Figure 1 shows the conical microchamber arrays (MCAs) fabrication process, SEM images of which are presented in Figure 1a,b. The method of forming MCAs included several stages.

At the first stage, a PDMS stamp, fixed on a glass slide, was used to make a patterned film from PLA chloroform solutions (1, 1.5, 2, and 2.5 wt %) by the dip-coating method. In this case, the film deposition uniformity was controlled by a constant extraction rate of the PDMS stamp, which was 1 mm/s.

At the second stage, the active drug—ceftriaxone—was loaded by applying the antibiotic powder and subsequent precipitation into microwells [48]. The removal of excess powder was carried out by the brush. This procedure resulted in the absence of the powder outside the wells of the template.

At the third stage, the resulting microcontainers were sealed by heating patterned and flat PLA films for 5 s at 80 °C. For this, flat PLA film (3.5 wt % in chloroform) was preliminarily prepared on a glass slide using a motorized thin-film applicator (Baker applicator gap 100 µm, 1 mm/s). After heat-fusion, the PDMS stamp was cooled to room temperature and detached from the film. Manufactured MCA films were 2.5 × 3.5 cm in size.

### 2.4. Release of Ceftriaxone from Microchamber Arrays and a Total Load of Drug

Ceftriaxone is a high water-soluble cephalosporin antibiotic. Its concentration can be measured by the absorption spectra analysis in the near-ultraviolet region by characteristic absorption bands at 240 and 275 nm [49]. The measurements were carried out in the long-wavelength absorption band (275 nm).

Absorption spectra of the antibiotic solutions in saline (0.9 wt % NaCl) were preliminarily obtained in the concentration range from 0.1 to 70 μg/mL (Figure S1a). According to the obtained data, a calibration curve was plotted (Figure S1b), which was used to determine ceftriaxone contents in the samples further.

To assess the prolonged release of ceftriaxone from MCAs, the prepared films were placed in saline and incubated at 37 °C with constant stirring (300 rpm) from 15 min to 264 h (11 days). The samples were transferred to clean saline at each time point.

The total load of ceftriaxone was estimated from the release data and calculated as the total amount of drug released until the release was completed.

### 2.5. In Vitro Study

*Staphylococcus aureus* ATCC 29213, taken from Saratov State Medical University Microbiology, virology, and immunology department collection, was used as test culture for this series of experiments. All manipulations were performed according to the standard methodical recommendations. Bacterial growth inhibition studies were conducted by the agar diffusion method on 90 mm plates with Mueller-Hinton Agar No. 2. The agar plates were inoculated with an 18-h bacterial culture, containing 1.5 × 10^8^ CFU/mL (McFarland 0.5). MCA samples 1.5 × 1.5 cm in size were placed on dried inoculated agar surface and incubated at 37 °C. We used two incubation schemes. To demonstrate the prolonged release of the antibiotic, we transferred the samples daily to new dishes with fresh bacterial culture and evaluated the antibacterial effect. In the second scheme, we assessed the influence of therapeutic ultrasound exposure on the MCA antibacterial effect. Ultrasound exposure was performed 3 h after the incubation started. In this case, we continued to incubate the samples on the same plates for seven days to demonstrate the effectiveness of inhibiting the growth of bacteria around the film for a long time. The antibacterial effect was counted by bacterial inhibition zones area. Ultrasound exposure was performed with Dynatron D125 Ultrasound Therapy Machine at 1 Mhz frequency, power of 2 W, for 1 min for each sample.

### 2.6. Characterization Technique

Scanning electron microscopy (SEM) measurements were performed with a VEGAIII (TESCAN, Czech Republic) microscope at an operating voltage of 5 kV. Before measurement, gold was deposited onto the sample (∼5 nm gold layer) using an Emitech K350 sputter-coater (Quorum Technologies Ltd., Ashford, UK).

Confocal Laser Scanning Microscopy (CLSM) measurements were performed with a Leica TCS SP8 X in reflection mode at a wavelength of 488 nm with 20x/0.7 n.a. objective lens. The sample was pre-covered with a thin layer of gold, similar to the pre-treatment for SEM measurements. To reconstruct the sample’s structure, the Z-stack was measured with a Z step less than the optical resolution of the microscope. Then, using the Gwyddion software package [50], the Z-stack was recalculated into a heightmap based on the maximum reflection intensity, and the radial profile of an individual microchamber averaged over the angle was calculated. If the reflection signal on the vertical walls of the microchamber was zero, these pixels were excluded from the averaging of the radial profiles over the angles.

Absorption spectra were recorded using a Shimadzu UV-1800 spectrophotometer (Shimadzu, Kyoto, Japan) in a standard 10-mm quartz cuvette.

## 3. Results and Discussion

### 3.1. Characterization of Microchamber Arrays Containing Ceftriaxone, the Total Load of Drug

All resulting MCAs had a conical shape with a height of 21 ± 1 µm (Figure 1a,b). However, changes in PLA concentration during patterned film formation resulted in a decrease in the usable volume of microcontainers and an improvement in retention.

**Figure 2:**
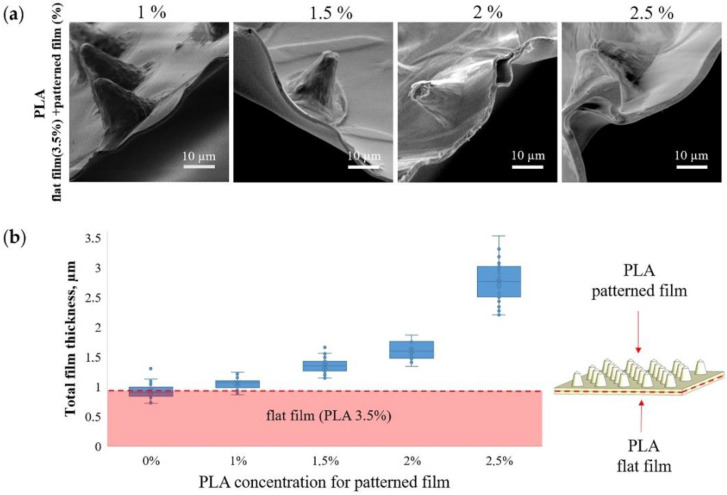
SEM images of microchamber arrays edge, obtained at different concentrations of PLA in the patterned film (**a**) and the dependence of the film thickness on the PLA concentration (**b**).

Figure 2a shows SEM images of the MCAs edge and contribution of patterned film thickness to total thickness (Figure 2b) at different PLA concentrations. When a patterned film is formed from 1 wt % PLA, its contribution to the total thickness is minimal and practically indistinguishable from the thickness of a flat film of 3.5 wt % PLA. In contrast, an increase in the PLA concentration to 2.5 wt % leads to an almost 3-fold increase in the total thickness. The PLA solution concentration is the most critical parameter that affects the PLA film thickness [51]. Therefore, it must be carefully chosen to synthesize microchamber arrays with different sizes, forms, and configurations [29].

**Figure 3:**
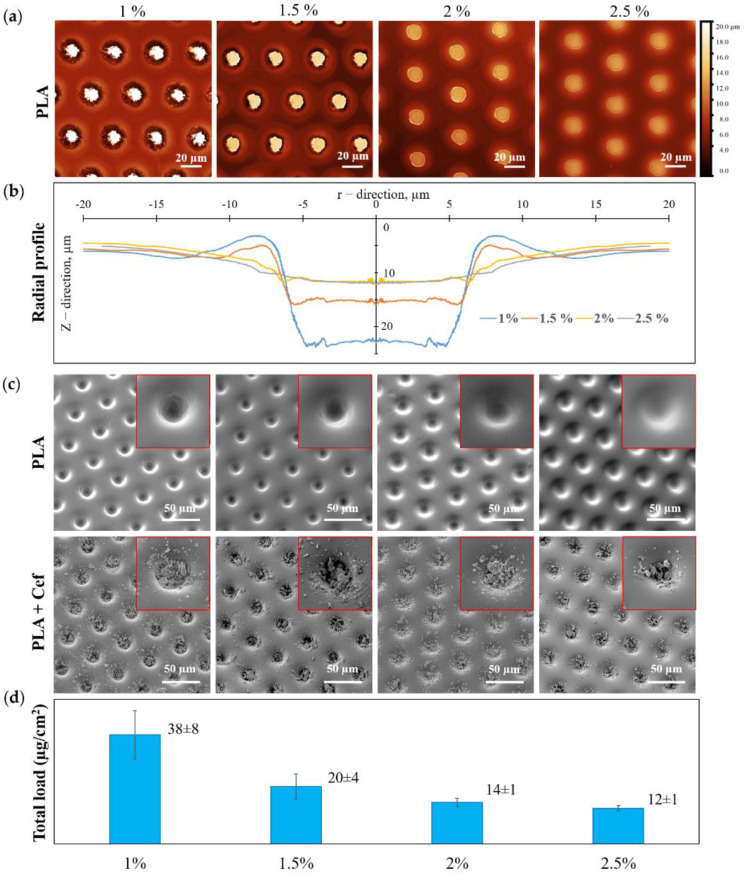
The dependence of the microwells shape and the microchambers loading efficiency with ceftriaxone on the initial PLA solution concentration: CLSM heightmap (**a**) and radial profile of the patterned film PLA (**b**)); SEM images of the patterned film of PLA (first row) and PLA with ceftriaxone (second row) (**c**); a total load of ceftriaxone in microchamber arrays (**d**).

An increase in PLA concentration during the formation of a patterned film led to a significant change in the morphology of microwells and, consequently, to a change in the loading drug efficiency (Figure 3). According to the CLSM data, Z-stack was recalculated into a heightmap based on the maximum reflection intensity (Figure 3a), and the radial profile of individual microwells averaged over the angle was calculated (Figure 3b). From the obtained radial profiles (Figure 3b) and SEM images for patterned films with different PLA concentrations (Figure 3c, first row), it can be seen that an increase in PLA concentration resulted in smaller microwells depth for loading. This fact can be explained by the initial solutions’ change in surface tension and a viscosity [52]. SEM images of patterned PLA film after drug loading are shown in Figure 3c (second row). This data reflects the change in the amount of loaded substance with increasing PLA concentration. A visual assessment of the SEM images shows that for the maximum PLA concentration, the load of the substance is the smallest, which entirely correlates with the data on the total load of MCAs.

The total drug loading of MCAs was calculated in terms of the samples area (Figure 3d). The total loading data demonstrates the highest drug loading in the patterned films of 1 wt % PLA, which was 38 ± 8 µg/cm^2^. With an increase in PLA concentration, the loading efficiency gradually decreases, and for a film of 2.5 wt % PLA, the loading was minimal and amounted to be 12 ± 1 µg/cm^2^.

### 3.2. Release of Ceftriaxone from Microchamber Arrays Depending on the Initial PLA Solution Concentration

**Figure 4:**
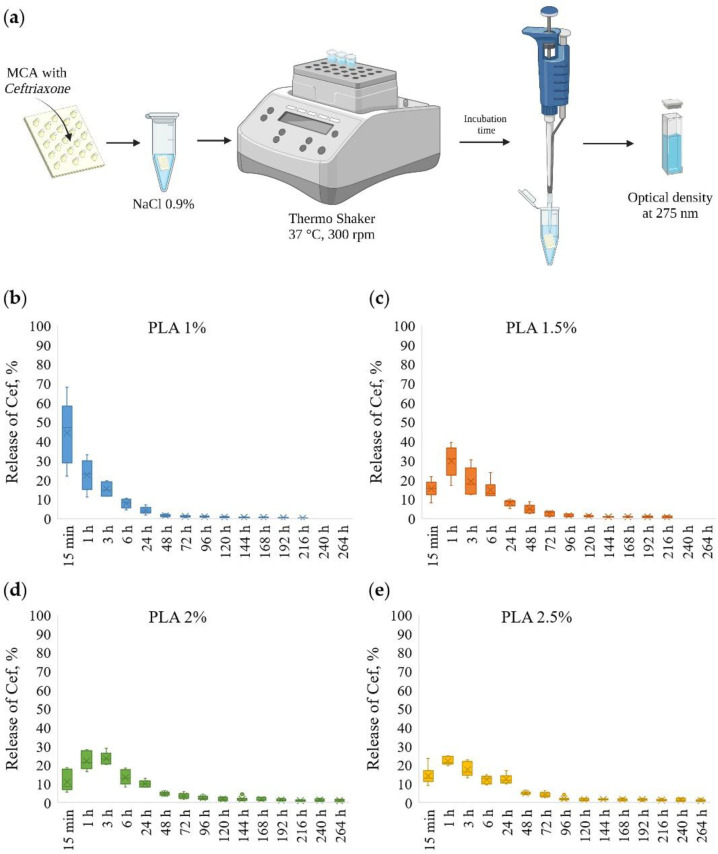
The scheme for the determination of prolonged release of ceftriaxone from microchamber arrays (**a**); prolonged release profiles of ceftriaxone from microchamber arrays prepared at various concentrations of PLA in a patterned film (from 1 to 2.5%) (**b**–**e**).

The scheme for the determination of kinetic of ceftriaxone prolonged release from MCAs is shown in Figure 4a. The ceftriaxone release profiles from MCAs prepared at various PLA concentrations are presented in Figure 4b–e and Figure S2.

The MCAs made out of 1 wt % PLA had a low holding capacity, and after 15 min, ~50% of loaded ceftriaxone was released. With further incubation for 6 h, another ~40% was eluted, and after 24 h, less than ~5% of the drug remained; thus, after 24 h, more than ~95% of the substance was released from the MCAs.

By changing PLA solutions from 1.5 to 2.5 wt %, the release of ceftriaxone slowed down. In the first 15 min, ~10–15% of a total load of ceftriaxone was released. With further incubation for 6 h, another ~60% of the total load was eluted, and after 24 h, about ~10% of the drug remained. Thus, after 24 h, ~80–85% of the drug was released from the MCAs. With this, the remaining amount of the drug continues to be released during subsequent incubation for 48 and 72 h. Another ~5% of the substance was released in 48 and 72 h.

### 3.3. Bacteriological Activity

**Figure 5:**
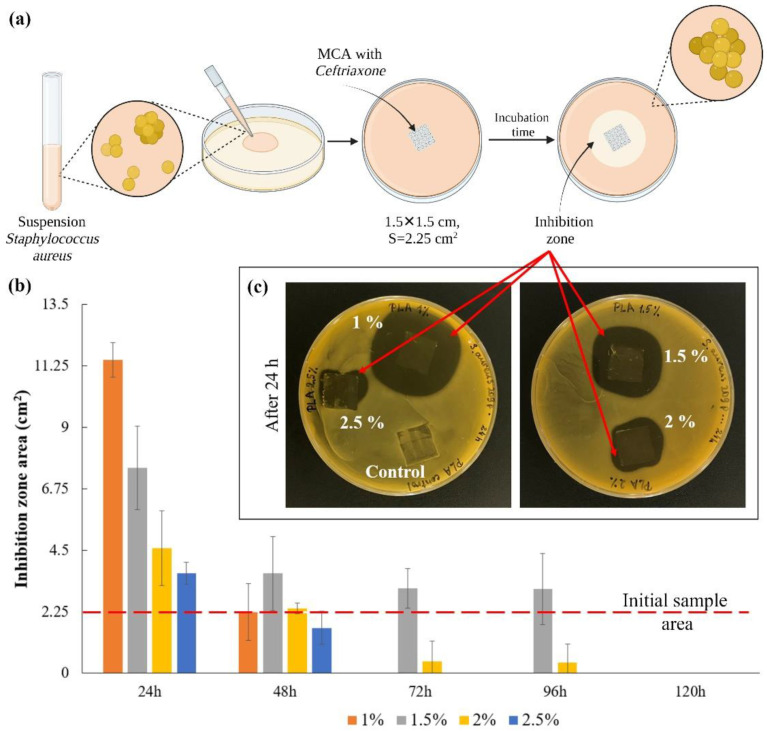
The scheme for assessing the bacterial activity of microchamber arrays containing ceftriaxone (**a**); the inhibition zones area after the first 24 h (**b**); the image of plates with samples after 24 h (**c**).

The scheme for assessing the bacterial activity of microchamber arrays containing ceftriaxone is shown in Figure 5a. In this series of experiments, the films were placed on an agar plate for 24 h and transferred to new plates after the next 24 h. The experiment was carried out for 5 days (Figure S3). The area of inhibition zones growth was counted every 24 h (Figure 5b). The image of plates with samples after the first 24 h are shown in Figure 5c. The control sample, to some degree, causes inhibition of bacterial growth, most likely due to mechanical restriction of oxygen access to the medium and acidification of the medium due to hydrolysis of the film polymer in a humid environment [53]. Quantitatively, only clearly distinguishable zones without bacterial growth were considered [54]. On the first day, the thinnest film of 1 wt % PLA gave the maximum size of the inhibition zone, but this effect faded after 48 h. For films with average polymer concentrations (1.5 and 2 wt %), this effect persisted for more than 72 h after the start of the experiment (Figure S3). This is primarily due to the fact that an increase in the viscosity of the medium in which the release takes place leads to a decrease in the rate of diffusion of the drug into the medium [55,56,57]. Due to this, the antibacterial effect on agar plates persisted longer than expected, according to the release data in saline. The investigation results show that the most effective PLA concentration is 1.5 wt %. This result correlates with the release results shown in Figure 4.

**Figure 6:**
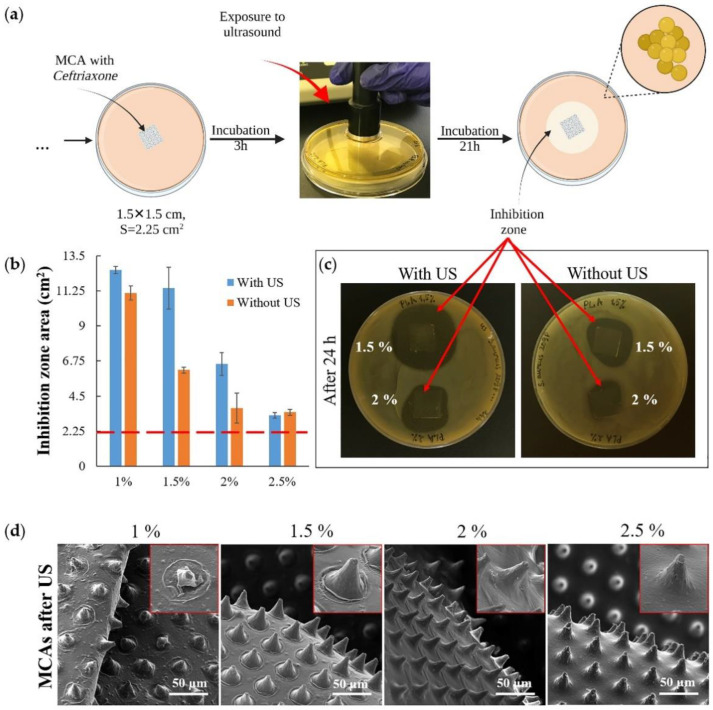
The scheme for assessing the bacterial activity of microchamber arrays containing ceftriaxone under exposure to ultrasound (1 Mhz frequency, 2 W power; 1 min for each sample) (**a**); the inhibition zones area 21 h after exposure to ultrasound (**b**); the image of plates with samples with and without exposure to ultrasound (**c**); SEM images of microchamber arrays containing ceftriaxone after exposure to ultrasound (insert size 40 × 40 μm) (**d**).

Another set of experiments was conducted to investigate the ultrasound (US) effect on releasing an antibacterial drug from MCAs (Figure 6a). Agar plates were inoculated with *S. aureus*, then the samples were placed on the plates and incubated at 37 °C for the next 3 h. Then the plates containing the samples were exposed to US (Figure 6a, exposure to ultrasound) and incubated for 21 h under the same temperature conditions. A control series of plates were set up with samples not exposed to US in parallel. After 24 h, the areas of bacterial growth inhibition zones in both series of samples were counted (Figure 6b). The result of the experiment shows that US exposure has little effect on the minimum (1 wt % PLA) and maximum (2.5 wt % PLA) thickness films that were used in the experiment; the effect was observed only for films with an average polymer concentration (1.5 wt % and 2 wt % PLA) (Figure 6c). For 2.5 wt % PLA, the exposure power was insufficient to damage the upper drug-containing layer (Figure S4 and Figure 6d, 2.5%), and for 1 wt % PLA, most of the drug had already been released by the time the US exposure began (Figure S4 and Figure 4b). The most significant effect was obtained with the 1.5 wt % PLA (Figure 6b,c) samples, which also showed the highest efficiency in the experiment described in Figure 5. Finally, it must be noted that ultrasound by itself did not have any antibacterial efficacy at the frequency that has been used. This is confirmed by the confluent growth of bacteria around the control MCA sample, which was exposed to ultrasound along with the samples containing the antibiotic (Figure S4).

The most significant changes in film morphology after ultrasound exposure were obtained for samples of 1 and 1.5 wt % PLA (Figure 6d). However, we did not find any reliably distinguishable micro-damages (microcracks) on the microchambers. Our previous results on a fluorescent cargo model also indicate that ultrasound exposure may not cause discernible damage to the microchamber shell when the cargo is fully released from it [32]. Ultrasound is not only a good trigger for opening microchambers, but also it increases the diffusion rate of the drug from the carrier [58,59]. At the same time, ultrasound-induced diffusion will proceed faster in liquid media.

Overall, our data show that low molecular weight loads retention efficiency was increased with increasing patterned film thickness, most likely associated with a decrease in the number of nano- and microdefects resulting from printing MCAs. At the same time, the release of the antibiotic was becoming longer and more uniform. However, a decrease in the total drug loading, with an increase of the film thickness to 1.82 ± 0.35 μm (2.5% PLA), did not allow the release of a ceftriaxone in sufficient amount to suppress the growth of *S. aureus* effectively. Thus, the optimum antibacterial activity of MCAs was observed for films with a thickness of 0.42 ± 0.12 μm. In addition, they contained more antibiotics than films with a thickness of 0.68 ± 0.15 μm (2% PLA), and released it more evenly, in comparison with films with a thickness of 0.13 ± 0.1 μm (1% PLA).

One of the main advantages of the microchamber fabrication process is the minimal effect on the cargo during the preparation; factually, it heats for 5 s only at 80 °C. As we have shown earlier, that approach allows us to preserve the activity of even poorly stable biological molecules such as adrenaline [32]. Our work focused on elaborating the approach to encapsulate ceftriaxone in sufficient amount and evaluating its release profile regarding variable thicknesses of microchamber walls. Thus, the antibacterial effect reported in work proved the efficient release and activity of ceftriaxone. Therefore, we have chosen one of the important test cultures (*S. aureus*) to explore these particular MCA fabrication aspects and confirm antibacterial activity. We expect that the developed biomaterials will have similar antibacterial activity for other Gram-positive and Gram-negative aerobic and some anaerobic bacteria sensitive to ceftriaxone [47] if MCAs are done using the same fabrication protocol.

In general, according to our data, we can conclude that such “smart films” with microchamber arrays are a promising biomaterial that can be used as dressing or coating to modify the surface of implants and stents [31,60]. In addition, it can allow the mechanical protection of damaged skin and tissues, prolonged and triggered antibiotic release.

## 4. Conclusions

This study demonstrates “smart” PLA-based films with microchamber arrays containing ceftriaxone to act as antibacterial biomaterial to inhibit the growth of *S. aureus*. Variation of PLA concentration from 1 to 2.5 wt % leads to a change in the patterned film thickness, antibiotic loading efficiency, release profile, and the antibacterial effect. The films formed from 1 wt % PLA have the lowest holding capacity and release ~50% of the total amount of the drug after 15 min. After 24 h in such containers, only ~5% of the drug remained. The films formed from higher concentrations (1.5, 2, and 2.5 wt % PLA) can retain the substance for a longer time. Their release profiles were similar and more uniform compared to 1 wt % PLA. In the first 15 min, they released only 10–15% of the total drug amount, and the release continued from them even after 48 and 72 h (~5%). Despite the similarity of the release profiles, the 1.5 wt % PLA film has the advantage of allowing more drug to be loaded at 20 ± 4 μg/cm^2^ versus 12 ± 1 μg/cm^2^ (2.5 wt % PLA). In vitro studies on *S. aureus* have shown that a 1.5 wt % PLA film retained its inhibitory effect on bacterial growth during 96 h. With the ultrasound-controlled release of the antibiotic from MCAs, the best result was also obtained for a film of 1.5 wt % PLA.

Thus, we demonstrate that film with MCAs provides a sufficient drug loading and could be used as an antibacterial coating for implantable medical devices, such as catheters, orthopedic structures. Moreover, it can be used as a bandage material. The latter is especially important for regions with a hot climate and poorly developed communication systems when it is necessary to prevent wound infection during patient transportation to the hospital. The ability to control the release profile using ultrasound exposure allows increasing the antibiotic’s concentration in case symptoms worsen locally. Combining prolonged (for prevention) and triggered (for treatment) antibiotic release can allow selecting the optimal therapeutic strategy, taking into account individual patient characteristics.

## Data Availability

Data underlying the results presented in this paper are not publicly available at this time but may be obtained from the authors upon reasonable request.

## Supplementary Materials

The following are available online at https://www.mdpi.com/article/10.3390/pharmaceutics14010042/s1, Figure S1: Absorption spectra of the ceftriaxone in saline were preliminarily obtained in the concentration range from 0.1 to 70 μg/mL (a); calibration curve (b); Figure S2: The overall prolonged release profile of ceftriaxone from microchamber arrays prepared at various concentrations of PLA in a patterned film (a) PLA 1%, (b) PLA 1.5%, (c) PLA 2% and (d) PLA 2.5%; Figure S3: Image of plates with samples: inhibition of bacterial growth with daily samples transferred to new plates during 5 days; Figure S4: Image of plates with samples after ultrasound exposure; without ultrasound exposure: inhibition of bacterial growth.
