# Supplementary material for: “Smart” Polylactic Acid Films with Ceftriaxone Loaded Microchamber Arrays for Personalized Antibiotic Therapy
Source: Pharmaceutics. 2021 Dec 26;14(1):42. doi: 10.3390/pharmaceutics14010042 (PMC8781070; doi:10.3390/pharmaceutics14010042)
Supplement: Supplementary file 1 [file pharmaceutics-14-00042-s001.zip › pharmaceutics-1503799-supplementary.pdf]

pharmaceutics

MDPI

Supplementary Materials: “Smart” Polylactic Acid Films with Ceftriaxone Loaded Microchamber Arrays for Personalized Antibiotic Therapy

Ekaterina A. Mordovina, Valentina O. Plastun, Arkady S. Abdurashitov, Pavel I. Proshin, Svetlana V. Raikova, Daniil N. Bratashov, Olga A. Inozemtseva, Irina Yu. Goryacheva, Gleb B. Sukhorukov and Olga A. Sindeeva

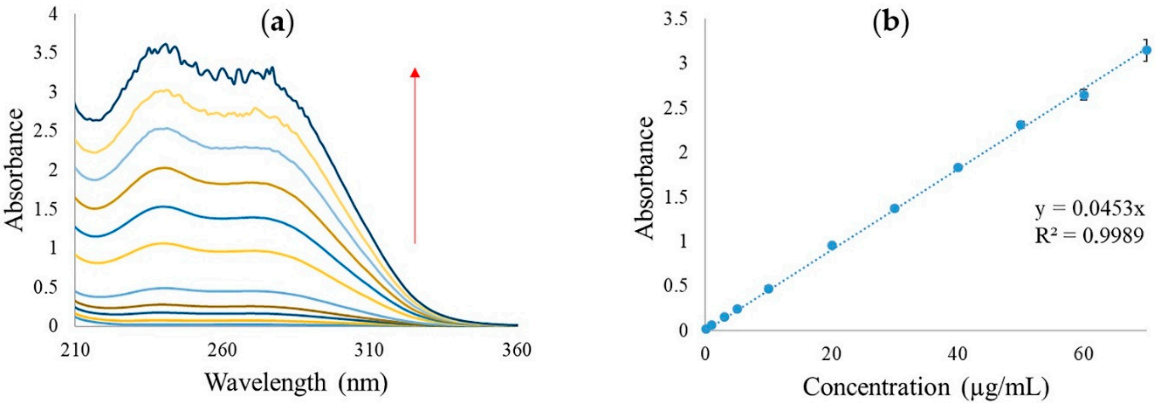

Figure S1. Absorption spectra of the Ceftriaxone in saline were preliminarily obtained in the concentration range from 0.1 to 70 μg/mL (**a**); calibration curve (**b**).

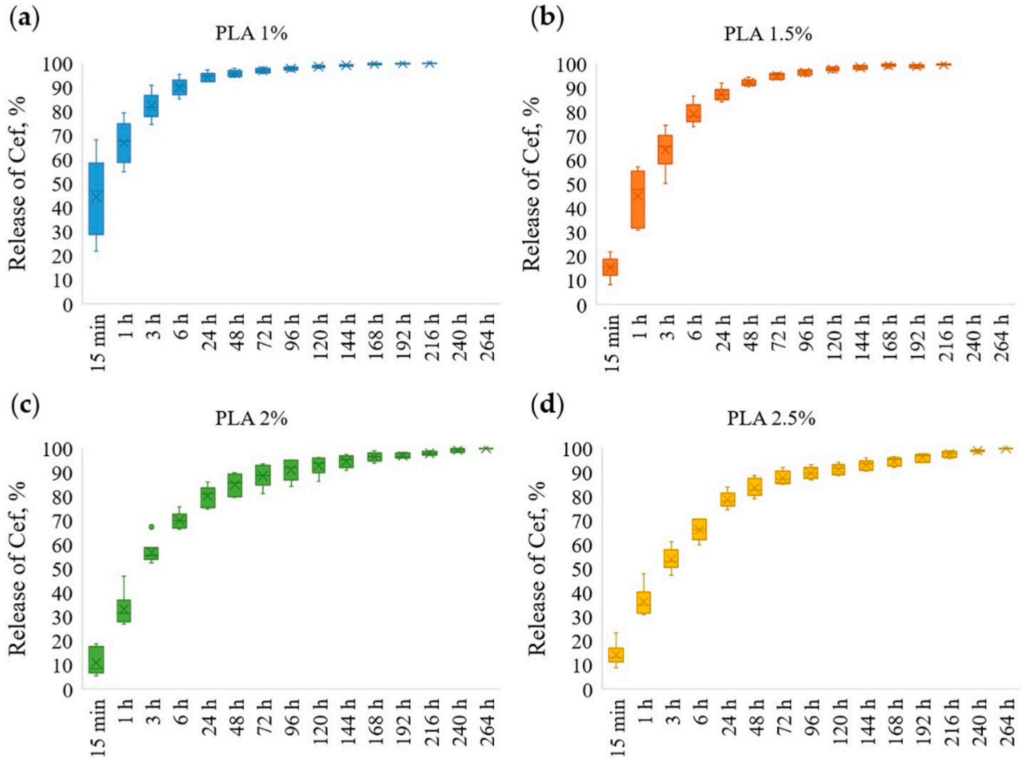

Figure S2. The overall prolonged release profile of ceftriaxone from microchamber arrays prepared at various concentrations of PLA in a patterned film: (**a**) PLA 1%, (**b**) PLA 1.5%, (**c**) PLA 2% and (**d**) PLA 2.5%.

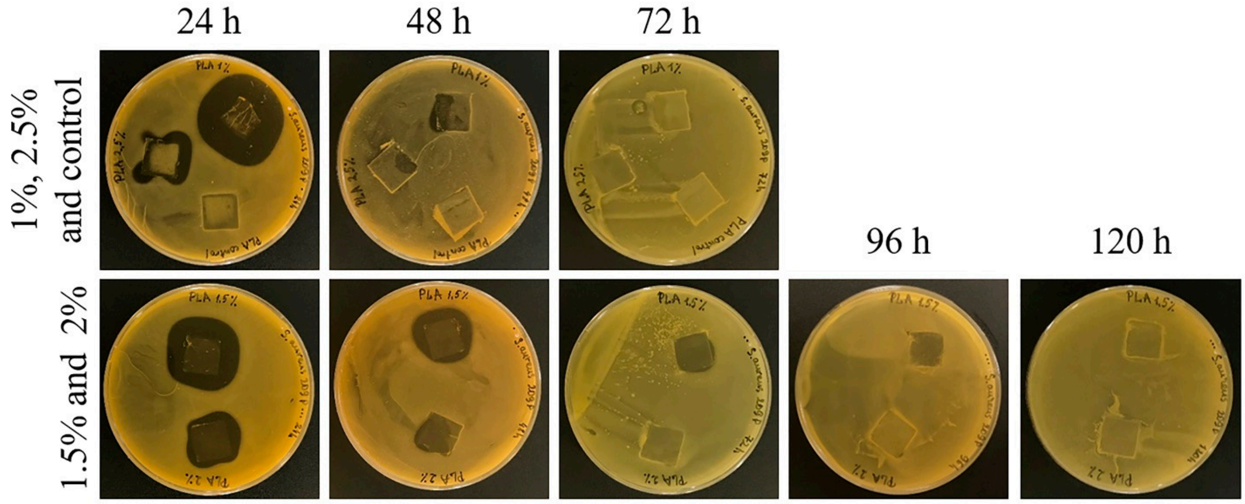

Figure S3. Image of plates with samples: inhibition of bacterial growth with daily samples transferred to new plates during 5 days.

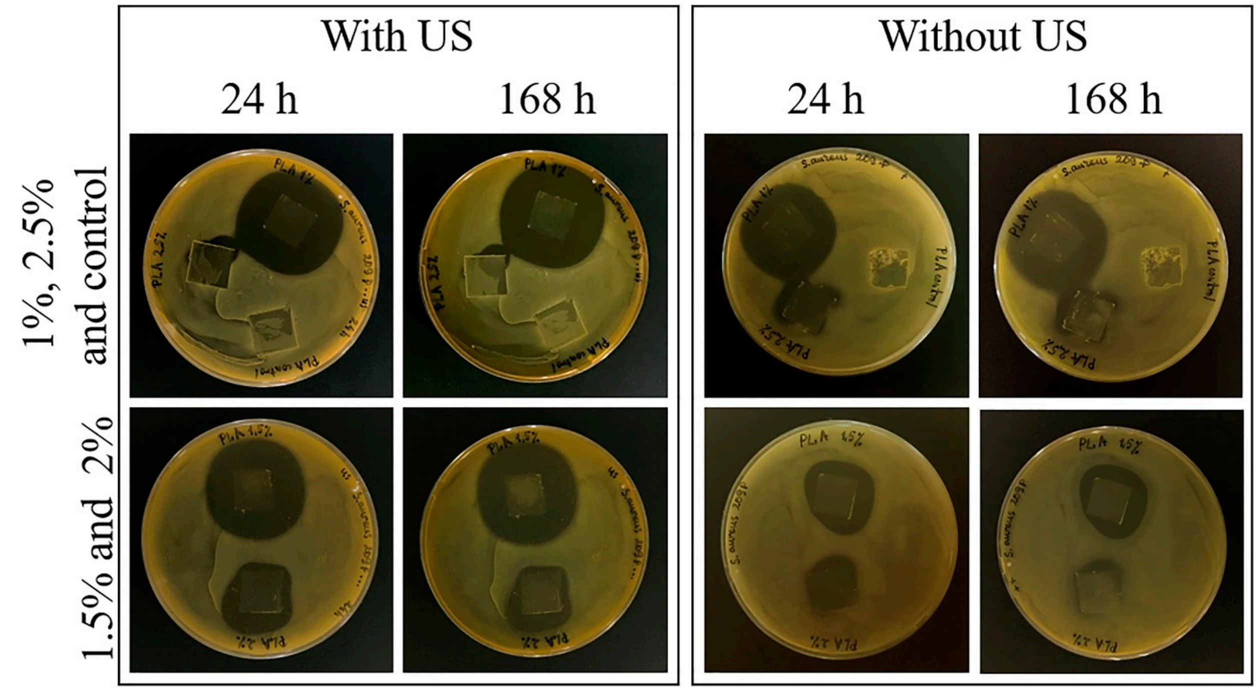

Figure S4. Image of plates with samples after ultrasound exposure; without ultrasound exposure: inhibition of bacterial growth.
